# Assistance Robotics and Biosensors 2019

**DOI:** 10.3390/s20051335

**Published:** 2020-02-29

**Authors:** Andrés Úbeda, Fernando Torres, Santiago T. Puente

**Affiliations:** 1 Human Robotics Group, Department of Physics, System Engineering and Signal Theory, University of Alicante, 03690 Alicante, Spain; 2 AUROVA Group, Department of Physics, System Engineering and Signal Theory, University of Alicante, 03690 Alicante, Spain; fernando.torres@ua.es (F.T.); santiago.puente@ua.es (S.T.P.)

**Keywords:** electromyographic (EMG) sensors, electroencephalographic (EEG) sensors, assistance robotics applications, robotic exoskeletons, robotic prostheses, advanced biomedical signal processing

## Abstract

This Special Issue is focused on breakthrough developments in the field of assistive and rehabilitation robotics. The selected contributions include current scientific progress from biomedical signal processing and cover applications to myoelectric prostheses, lower-limb and upper-limb exoskeletons and assistive robotics.

## 1. Introduction

In recent years, the use of robotics to help motor-disabled people has experienced a significant growth, mostly based on the development and improvement of biosensor technology and the increasing interest in solving accessibility and rehabilitation limitations in a more natural and effective way. For this purpose, biomedical signal processing has been combined with robotic technology, such as exoskeletons or assistive robotic arms or hands. However, efforts are still needed to make these technologies affordable and useful for end users, as current biomedical devices are still mostly present in rehabilitation centers, hospitals and research facilities. This Special Issue covers several of the recent advances in robotic devices applied to motor rehabilitation and assistance.

## 2. Contributions

The Special Issue has collected eight outstanding papers covering different aspects of assistance robotics and biosensors. The selected contributions cover several main topics related to assistance robotics, from the control of myoelectric prostheses to the rehabilitation and assistance of the lower and upper limbs. What follows is a brief summary of the scope and main contributions of each of these papers, provided as a teaser for the interested reader.

Upper-limb transradial amputation is cause of severe disability, and a key technology to solve this issue is the use of prosthetic devices for motor substitution. A particular way of controlling these devices is through electromyographic (EMG) signals. In [1], a low-cost non-intrusive myoelectric control is proposed by applying gated recurrent neural networks to data collected from a MYO Armband. This study achieves a 77.85% accuracy when classifying six different hand movements with a generalized classification model.

Other issues that affect classification accuracy are evaluated in [2,3]. An adaptive classification model based on directional forgetting is proposed in [2]. This novel algorithm addresses signal instability issues through a calibration of the model in time, showing good results in a small number of volunteers. Another key factor in myoelectric control is the introduction of an adequate force feedback for the prosthesis. In [3], vibrotactile actuators are used to assess the optimal force feedback patterns delivered to the user. This study reflects that changes in amplitude and frequency level do not show significant differences in the discrimination of vibration patterns. On the other hand, a reduced number of vibration levels increases detection accuracy. Authors propose this study as a starting point for the future optimization of training protocols and for the evaluation of the location and number of vibrotactile actuators.

The rehabilitation of the lower limb has been addressed using a variety of devices, from exoskeletons to walkers. Lower-limb exoskeletons are mainly used in gait rehabilitation. HYBRID is an ambulatory robotic gait trainer with movement induction and partial weight support [4]. This device combines a conventional lower-limb exoskeleton, H1, with an active partial body weight support system (PBWS) to improve locomotive capabilities and minimize muscular effort. This device has proven to be feasible in future clinical applications. Another approach to walking assistance is AGORA [5]. It is a robotic walker (smart walker) that includes several system modules: navigation, human detection, safety, user interaction and social interaction. All these modules are ruled by shared control strategies that command the correct ambulation of the patient.

In [6], the authors propose a method to detect the appearance of sudden obstacles from electroencephalographic (EEG) signals. This method could be applied in the supervision of gait rehabilitation using exoskeletons. The EEG data collected from healthy subjects was processed and classified using linear discriminant analysis, showing an average detection rate of obstacle appearance of about 63.9%, with a false positive rate of 2.6 obstacles per minute. These results were a promising improvement compared to previous studies.

In upper-limb rehabilitation, the physiological interaction of the patient with the robotic device is critical. In [7], two different physiological control methods based on EEG and electrooculography (EOG) are evaluated, while measuring stress levels from skin conductance level (SCL) and heart rate variability (HRV). This study shows that EEG control is associated with a higher level of stress and mental workload when compared to EOG control. An alternative human-robot interaction method is shown in [8]. AMICUS is a head motion-based interface for the control of an assistive robot. In this study, the device is tested by both healthy and tetraplegic participants to perform pick-and-place tasks. The results show that the head motion control is smooth and precise, deriving a high user acceptance.
